# Detection of *Giardia* and *Cryptosporidium* in surface water of a subarctic city

**DOI:** 10.1016/j.fawpar.2025.e00262

**Published:** 2025-04-11

**Authors:** Christina A. Ahlstrom, Michael P. Carey, Damian M. Menning, Jonathan A. O'Donnell, Andrew M. Ramey

**Affiliations:** a U.S. Geological Survey Alaska Science Center, Anchorage, Alaska, United States of America; b National Park Service, Arctic Network, Anchorage, Alaska, United States of America

**Keywords:** Giardia, Cryptosporidium, One health, Alaska

## Abstract

*Giardia* and *Cryptosporidium* spp. are globally distributed protozoan parasites that can cause gastrointestinal disease in humans and animals. These zoonotic parasites and their ecological relationships have been understudied in Alaska and elsewhere, despite being identified as priority zoonotic pathogens. We aimed to detect and characterize *Giardia* and *Cryptosporidium* spp. in waterbodies within Anchorage, Alaska, USA using two methods, including the Environmental Protection Agency (EPA) Method 1623 that relies on microscopy and a molecular detection approach. The molecular approach was ultimately unsuccessful and therefore only data obtained using Method 1623 are presented. *Giardia* or *Cryptosporidium* spp. was detected from nine of 15 urban streams and lakes sampled (60 %), six of which were positive for both parasites (40 %). Fewer than 10 cysts or oocysts were detected in 10 L of surface water. Further research to characterize *Giardia* and *Cryptosporidium* beyond the genus level would help elucidate the zoonotic potential and ecology of these parasites within the region and more broadly in Alaska.

## Highlights

• We sampled surface water for *Giardia* and *Cryptosporidium* spp. in sub-Arctic Alaska.
• Low levels of one or both parasites were detected in 60 % of waterbodies sampled.
• Further research could elucidate zoonotic potential of parasites in this region.

## 1 Introduction

*Cryptosporidium* and *Giardia* spp. are widespread zoonotic protozoan parasites that can cause gastrointestinal illness in numerous hosts, including wildlife, livestock, companion animals, and humans. Despite their extensive geographical distributions and broad host ranges, these parasites and their ecological relationships have generally been understudied, particularly in Arctic and sub-Arctic regions (Jenkins et al., 2013; Robertson and Debenham, 2022). Infection with *Giardia* or *Cryptosporidium* spp. can cause diarrhea and, in severe cases, be fatal in humans and domestic animals; however, the clinical effects on wildlife hosts are largely unknown (Appelbee et al., 2005). Humans and animals are known reservoirs of zoonotic species of both *Cryptosporidium* and *Giardia*, though not all species of these parasites are zoonotic and some appear to be host specific (Appelbee et al., 2005; Hunter and Thompson, 2005). Molecular characterization at the species, assemblage, or sub-assemblage level is recommended to better understand the risks to human and animal health, and the ecology and transmission of these parasites (Thompson and Ash, 2016). For example, high-resolution genetic data for *Giardia duodenalis* at the assemblage and sub-assemblage level has proven useful for evaluating parasite transmission via contaminated water between wild animals and humans (Tsui et al., 2018).

In 2019, a Centers for Disease Control and Prevention One Health Zoonotic Disease Prioritization for Multisectoral Engagement in Alaska workshop identified both *Cryptosporidium* and *Giardia* spp. as priority zoonotic pathogens (https://www.cdc.gov/one-health/media/pdfs/alaska-508.pdf?CDC_AAref_Val=https://www.cdc.gov/onehealth/pdfs/Alaska-508.pdf, accessed 19 November 2024) given prior findings that human exposure to *Giardia* and *Cryptosporidium* spp. may be relatively high among Alaska residents and rates of giardiasis are consistently among the highest in the United States (Jenkins et al., 2013; McLaughlin and Castrodale, 2023; Miernyk et al., 2019; Painter et al., 2015; Yoder et al., 2010). The ongoing range expansion of beavers into northern Alaska (Tape et al., 2018) may also increase risk of infection with these and other zoonotic disease agents due to ecological shifts and landscape disruption (Jenkins et al., 2013). However, there has been limited sampling for these parasites in terrestrial and semi-aquatic freshwater wildlife (Jenkins et al., 2013) or the physical environment (Norman et al., 2013) throughout Alaska. In Alaska, *Cryptosporidium* or *Giardia* spp. has been detected in some terrestrial (e.g., Arctic fox, caribou) and marine species (e.g., polar bear, bowhead whale, ringed seal, harbor seal) (Hueffer et al., 2011; Hughes-Hanks et al., 2005; Siefker et al., 2002; Van Hemert et al., 2023), though the ecology and spatial distribution of these parasites are poorly understood.

In 1999, the U.S. Environmental Protection Agency (EPA) published a standardized protocol, Method 1623, to detect and quantify *Giardia* and *Cryptosporidium* spp. in water samples (Environmental Protection Agency, 2012), which is now commonly used for the detection of these parasites globally (Ongerth, 2013). It has been recommended to use a microscopic method (e.g., Method 1623) in tandem with molecular methods for characterization of these parasites, as molecular methods alone have demonstrated inconsistent detection rates (Thompson and Ash, 2016). In this study, we aimed to use two methods to detect *Giardia* and *Cryptosporidium* spp. from surface waters within the city of Anchorage, Alaska to inform future research on the epidemiology of these parasites in Arctic and sub-Arctic regions. Approaches included Method 1623, employing microscopy, as well as a molecular method, based on membrane filter dissolution and qPCR. The molecular approach was unsuccessful and therefore only data obtained using Method 1623 are presented.

## 2 Materials and methods

### 2.1 Detection using Method 1623

Fifteen waterbodies within the municipality of Anchorage were selected for surface water sampling, representing both lake (*n* = 11) and creek (*n* = 4) water. Sampling sites were located within municipal parks and were characterized as “highly residential” if five or more houses bordered the waterbody. Samples were collected July 12 – September 20, 2021. A total of 10 L of surface water was filtered on-site through an Envirochek HV capsule (Pall Corporation, NY) for each replicate, according to Method 1623 (Environmental Protection Agency, 2012). Three replicates were filtered at each site using a Geopump Series II Peristaltic Pump (Geotech Environmental Equipment, Inc., Denver, CO) and limiting flow orifice to maintain a flow rate less than 2 L per minute. A fourth “field duplicate sample” was filtered in the same way to determine parasite recovery from filter capsules. Filter capsules (a total of four capsules per site) were kept cool on ice until sent to an EPA certified laboratory (Lab/Cor, Inc., Seattle, WA) for detection and quantification of *Cryptosporidium* oocysts and *Giardia* cysts.

Samples were eluted less than 96 h after sample collection and processed according to Method 1623 (Environmental Protection Agency, 2012). The field duplicate was spiked with a known concentration of gamma-irradiated *Cryptosporidium* and *Giardia* and was processed using the same preparation and analysis techniques as the field samples. Briefly, the eluant was concentrated using centrifugation where all but 5 mL of supernatant and pellet were removed. Immunomagnetic antigen-antibody separation was then performed on the remaining pellet (≤ 0.5 mL) in order to separate the organisms of interest from non-specific interferences. Well slides were prepared for each individual sample in addition to a positive and negative control. Cysts and oocysts were then identified using fluorescein isothiocyanate (FITC), 4′,6-diamidino-2-phenylindole (DAPI), and differential interference contrast (DIC) microscopy. Cyst and oocyst counts as well as percent recovery at each location were recorded. Estimated cyst/oocyst counts were determined by calculating the number of cysts and oocysts expected based on percent recovery from the matrix spike filters.

### 2.2 Detection using molecular methods

At each site described above, 10 L of water was filtered through a 1.2 μm membrane filter (MilliporeSigma, Burlington, VT) for each of three replicates. As a positive control, 10 L of distilled water was spiked with 300*C. parvum* oocysts and 300 *G. lamblia* cysts (BioPoint Pty Ltd., Sydney, Australia) and subsequently filtered through a 1.2 μm membrane filter (MilliporeSigma, Burlington, VT). All samples were kept cool on ice until further processing. In the laboratory, filters were processed following a modified filter dissolution method described by Aldom and Chagla (1995). Filters were transferred into 50 mL conical tubes with 45 mL of acetone. After two minutes, tubes were centrifuged at 650 *g* for 15 min. The supernatant was removed, followed by successive additions and centrifugations of acetone, 95 % ethanol, and 70 % ethanol. The final pellet was resuspended in 10 mL of distilled water, which was then subjected to immunomagnetic separation using Dynabeads GC-combo (Applied Biosystems, Foster City, CA) following manufacturers' instructions. DNA was extracted from cysts and oocysts using Protocol 8B, described by Adamska et al. (2010). This method employes three cycles of boiling and freezing in liquid nitrogen followed by use of the QIAamp DNA Tissue Mini Kit (Qiagen, San Diego, CA). Samples were eluted in 50 μL elution buffer. Using qPCR, we attempted to amplify specific regions of the 18S rDNA gene in *Cryptosporidium* spp. (Hadfield et al., 2020) and *Giardia* spp. (Jothikumar et al., 2021). Genomic DNA from *Cryptosporidium hominis* and *Giardia intestinalis* (ATCC, Manassas, VA) were included in PCR reactions as positive controls and nuclease-free water was included as negative controls.

## 3 Results

### 3.1 Detection using Method 1623

**Fig. 1:**
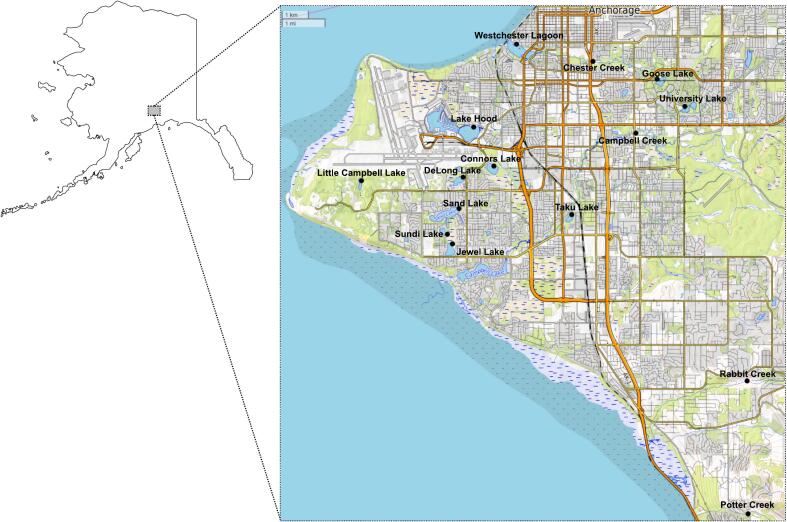
Map of Alaska with the inset showing 15 sites in the municipality of Anchorage where filtered surface water samples were collected between July and September 2021. The map was created using OpenStreetMap (www.openstreetmap.org). Grey shading indicates a residential and industrial areas and green shading indicates parklands and forested areas. (For interpretation of the references to colour in this figure legend, the reader is referred to the web version of this article.)

**Fig. 2:**
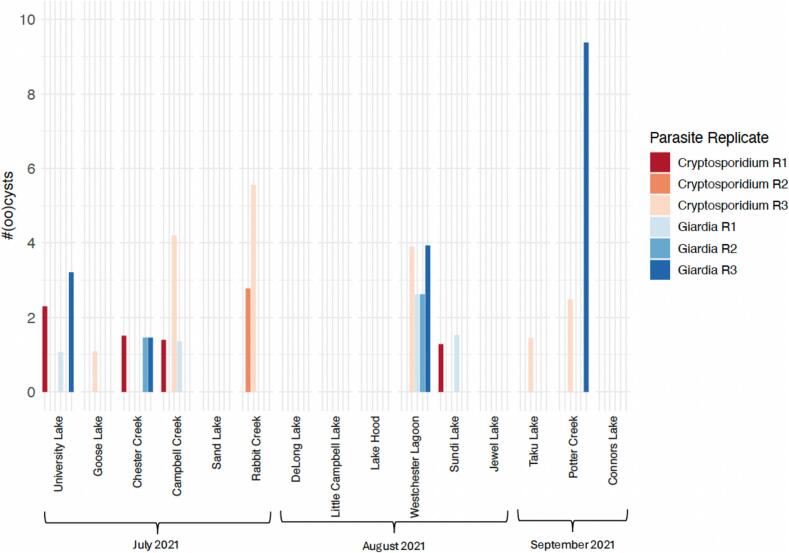
Bar chart indicating the estimated number of *Cryptosporidium* oocysts (red) and *Giardia* cysts (blue) from each of three 10 L replicates (R1–R3; represented by different shades) of filtered surface water at each site. (For interpretation of the references to colour in this figure legend, the reader is referred to the web version of this article.)

We sampled for *Cryptosporidium* oocysts and *Giardia* cysts in 15 waterbodies in the municipality of Anchorage (Fig. 1). Using Method 1623, we detected *Cryptosporidium* or *Giardia* spp. in at least one of three replicates from nine (60 %) and six (40 %) of these 15 waterbodies, respectively (Table 1; Fig. 2; Ahlstrom et al., 2025). All six sites at which *Giardia* spp. were detected were also positive for *Cryptosporidium* spp. Three sites were positive for only *Cryptosporidium* spp. (20 %) and six sites were negative for both parasites (40 %). All four creeks sampled in this study were positive for *Cryptosporidium* spp. and three were positive for *Giardia* spp. (Fig. 2). *Cryptosporidium* oocysts were either detected in a single replicate (*n* = 6) or two replicates (*n* = 2), whereas *Giardia* cysts were detected in a single replicate (*n* = 3), two replicates (n = 2), or all three replicates (n = 1). Aside from detection of *Cryptosporidium* spp. from all flowing waters sampled, there were no obvious waterbody characteristics (e.g., adjacency to dog parks or residential housing) that were associated with presence/absence of either parasite.

**Table 1:** Detections of *Giardia* cysts and *Cryptosporidium* oocysts in three replicates of 10 L filtered water samples from 15 urban lakes and creeks in Anchorage, AK, USA using EPA Method 1623. Counts are estimated based on percent recovery of matrix spike samples.

		*Cryptosporidium*			*Giardia*		
Location	Residential	Rep 1	Rep 2	Rep 3	Rep 1	Rep 2	Rep 3
Campbell Creek	Low	1.4	0	4.2	1.36	0	0
Chester Creek	Low	1.51	0	0	0	1.46	1.46
Connors Lake	Low	0	0	0	0	0	0
DeLong Lake	High	0	0	0	0	0	0
Goose Lake	Low	0	0	1.09	0	0	0
Jewel Lake	High	0	0	0	0	0	0
Lake Hood	High	0	0	0	0	0	0
Little Campbell Lake	Low	0	0	0	0	0	0
Potter Creek	Low	0	0	2.48	0	0	9.38
Rabbit Creek	Low	0	2.78	5.56	0	0	0
Sand Lake	High	0	0	0	0	0	0
Sundi Lake	High	1.28	0	0	1.53	0	0
Taku Lake	Low	0	0	1.45	0	0	0
University Lake	Low	2.3	0	0	1.07	0	3.21
Westchester Lagoon	High	0	0	3.9	2.62	2.62	3.93

*Cryptosporidium* and *Giardia* spp. were detected from sites sampled during all three months of surface water collection from both lakes and creeks (Fig. 2). The estimated number of *Cryptosporidium* oocysts and *Giardia* cysts in 10 L of surface water, after correcting for percent recovery, ranged from 0 to 5.6 oocysts and 0–9.4 cysts, respectively (Fig. 2). The recovery efficiency of parasites from filters was calculated by spiking a known number of parasites into a fourth field control filter collected at each site and quantifying the number of parasites recovered from each filter. Percent recovery of matrix spiked samples ranged from 38 % – 91 % for *Cryptosporidium* oocysts and 46 % – 93 % for *Giardia* cysts, though percent recovery for parasites from the same waterbody were generally within approximately 10 % with few exceptions (Supplemental Fig. 1).

### 3.2 Detection using molecular methods

Positive control genomic DNA generated a positive detection using qPCR and negative controls were negative. However, the positive control extract generated from distilled water spiked with *Giardia* and *Cryptosporidium* spp. failed to amplify in the qPCR reaction. Despite troubleshooting, we were unable to identify which step or steps failed in our molecular protocol. Thus, results describing detection using this approach are not presented.

## 4 Discussion

This study provides new information regarding the occurrence of *Cryptosporidium* and *Giardia* spp. in urban, sub-Arctic waterbodies. Given that all samples were collected by accessing waterbodies through municipal parks, waterbodies may have been inhabited or visited by diverse urban wildlife (e.g., beavers, river otters, waterfowl, moose), domestic dogs, and humans, all of which may harbor *Cryptosporidium* and *Giardia* spp. (Appelbee et al., 2005). Although livestock are often considered sources of *Cryptosporidium* and *Giardia* spp. (Budu-Amoako et al., 2011), Anchorage has no commercial animal agriculture. In 2011, a similar study detected *Cryptosporidium* spp. in 100 % and *Giardia* spp. in 67 % of river water collected within and adjacent to the municipality of Anchorage with up to 94 oocysts and up to 23 cysts in 10 L samples (Norman et al., 2013). We detected lower numbers of oocysts and cysts in our study, though found a comparable proportion of creeks positive for *Cryptosporidium* spp. (100 %) and *Giardia* spp. (75 %). The number of cysts and oocysts detected in our study was also comparably low relative to results reported from a watershed that spans both rural and urban land use in British Columbia (Prystajecky et al., 2014) and elsewhere (Gallas-Lindemann et al., 2013; Kumar et al., 2016; Ligda et al., 2020). Given the low numbers of cysts/oocysts detected in this study, these urban waterbodies are unlikely to pose significant risks to animal and human health (Okhuysen et al., 1999; Rendtorff, 1954).

Despite the comparatively lower number of oocysts and cysts detected in this study, our recovery efficiencies were similar or higher than percent recovery reported in previous studies of surface water (Ligda et al., 2020; Norman et al., 2013). The efficiency of parasites recovered from filters using Method 1623 has been shown to vary over time and space and may be influenced by the turbidity of the water undergoing filtration (DiGiorgio et al., 2002; Ongerth, 2013). We did not take water quality measurements, though water characteristics (e.g., turbidity) might partially explain differences in recovery efficiencies between sites in Anchorage as well as among studies (Ongerth, 2013).

Some *Giardia* and *Cryptosporidium* spp. are host-adapted to specific wildlife taxa and pose little risk to human and animal health, while others can infect a broad range of taxa, including humans (Appelbee et al., 2005). Thus, further genetic characterization of *Cryptosporidium* and *Giardia* spp. in Alaska surface water and wildlife is warranted to better understand the occurrence and distribution of those species and assemblages of highest zoonotic concern. Unfortunately, our attempt to apply molecular methods to determine *Cryptosporidium* and *Giardia* species or assemblages was unsuccessful. We were unable to identify which step failed in our protocol, as filter dissolution, immunomagnetic separation, and DNA extraction each involved multiple sub-steps. Cysts and oocysts may have been washed away during filter dissolution or immunomagnetic separation, or DNA extraction may have failed. Alternative methods, such as bead beating of the filter membrane, could be explored in future work to establish higher taxonomic resolution using molecular tools.

The following are the supplementary data related to this article.

Supplementary Fig. 1 Chart indicating the percent of spiked *Cryptosporidium* oocysts (red) and *Giardia* cysts (blue) recovered from field duplicate filters at each of 15 waterbodies sampled. Supplementary Fig. 1

## CRediT authorship contribution statement

**Christina A. Ahlstrom:** Conceptualization, Methodology, Validation, Formal analysis, Investigation, Resources, Data curation, Writing – original draft, Writing – review & editing, Visualization, Supervision, Project administration, Funding acquisition. **Michael P. Carey:** Conceptualization, Methodology, Investigation, Resources, Writing – review & editing, Project administration, Funding acquisition. **Damian M. Menning:** Conceptualization, Methodology, Resources, Writing – review & editing, Project administration, Funding acquisition. **Jonathan A. O'Donnell:** Conceptualization, Methodology, Resources, Writing – review & editing, Project administration, Funding acquisition. **Andrew M. Ramey:** Conceptualization, Methodology, Investigation, Resources, Writing – review & editing, Supervision, Project administration, Funding acquisition.

## Declaration of competing interest

No conflict of interest declared.

## Data Availability

Data are available in Ahlstrom et al. (2025).
