# Supplementary figures and images for: Detection of Giardia and Cryptosporidium in surface water of a subarctic city
Source: Food Waterborne Parasitol. 2025 Apr 11;39:e00262. doi: 10.1016/j.fawpar.2025.e00262 (PMC12032176; doi:10.1016/j.fawpar.2025.e00262)

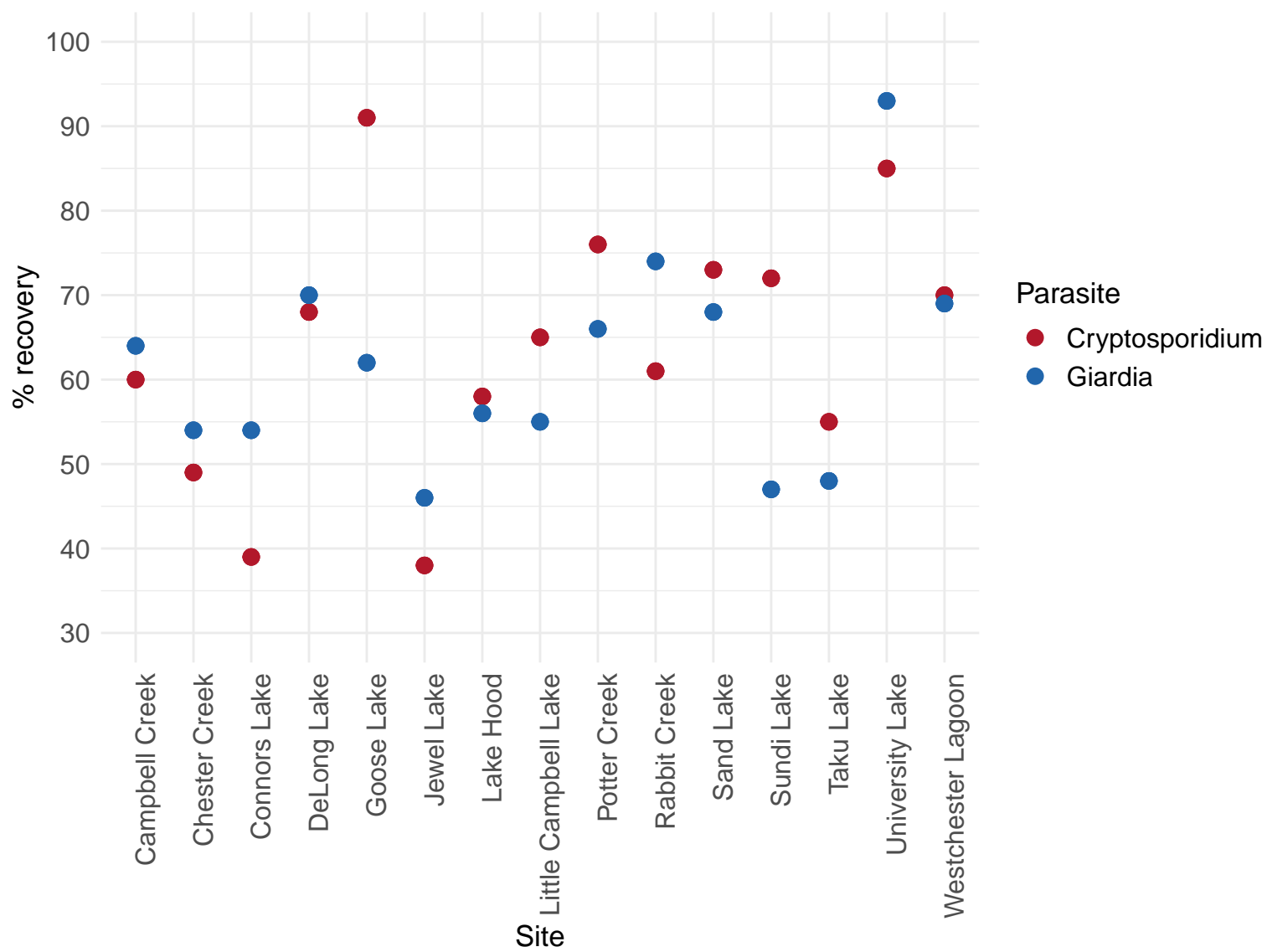

Supplement: Supplementary Fig. 1 — Chart indicating the percent of spiked Cryptosporidium oocysts (red) and Giardia cysts (blue) recovered from field duplicate filters at each of 15 waterbodies sampled. [file mmc1.pdf]
